# Comprehensive analysis of bony scapula morphology and anthropometry in a homogeneous population

**DOI:** 10.1186/s12880-025-01793-z

**Published:** 2025-07-02

**Authors:** Amr Elshahhat, Yehia Basyoni, Khaled Nour

**Affiliations:** Mansoura University, Mansoura, Egypt

**Keywords:** Acromioglenoid, Coracoacromial, CT, Glenoid index, Morphology, Scapula

## Abstract

**Background:**

The shoulder joint has consistently drawn the interest of radiologists, physiologists, and orthopaedic surgeons. The precise measurements and geometry of the scapula are crucial to understanding shoulder pathomechanics. It is necessary to understand typical variations in the glenoid, coracoid, and acromion to maximize shoulder procedures’ success. This study reports the morphological characteristics and anthropometric measurements of the scapular bone structures in a representative sample of our community.

**Methods:**

A total of 60 dry human scapulae were studied. Morphological variations in the suprascapular notch, acromion, and glenoid cavity were observed. The dimensions of the scapular body, glenoid cavity, and coracoid and acromion processes were measured.

**Results:**

When comparing the three-dimensional computed tomography (3D-CT) and sliding vernier calliper calibrations, no discernible variation was found in any of the measured parameters. The most prevalent morphologies were the oval-shaped glenoid, type II acromion, and type III suprascapular notch, corresponding to incidences of 50%, 70%, and 35%, respectively. The mean glenoid index among the determined parameters was 70% ± 1%. The average acromial thickness was 7.6 ± 1.4 mm, and the average coracoid tip-infer glenoid tubercle distance was 35 ± 5 mm.

**Conclusions:**

The scapular bony components exhibit significant interpopulation variation in their morphological and anthropometric parameters. 3D-CT scans and direct measurements yielded closely aligned results, confirming the accuracy of CT for scapular evaluation. This highlights the usefulness of CT in different shoulder interventions. Preoperative planning to ascertain the scapular bony dimensions and knowledge of the morphology of scapular components is always advantageous.

**Trial registration:**

Not applicable for this study.

**Supplementary Information:**

The online version contains supplementary material available at 10.1186/s12880-025-01793-z.

## Background

The scapula is a cornerstone of upper limb biomechanics, providing essential support and articulation for shoulder motion. Its intricate bony architecture not only stabilizes the shoulder girdle but also serves as an origin for numerous muscles and ligaments involved in upper limb function [1, 2]. Anthropometric assessment of scapular features is increasingly important in radiological and orthopaedic planning, especially for shoulder procedures such as arthroplasty, fracture fixation, and nerve decompression [3].

Preoperative planning is arguably more critical in shoulder arthroplasty than in other joint replacements due to the complexities of glenoid morphologies and the relatively small glenoid size. Moreover, only a limited portion of the scapula is accessible intraoperatively [4]. Morphological variations of the scapula have well-documented clinical consequences. For instance, suprascapular notch shape can influence the risk of nerve entrapment, particularly in narrow or U-shaped notches [5, 6]. The acromion type has also been associated with rotator cuff (RC) disease, with the hooked variant linked to higher rates of impingement and tendon tears [7]. Additionally, accurate preoperative assessment of the coracoid process is crucial in procedures such as the Latarjet, where coracoid size may predict the success of converting off-track Hill-Sachs lesions to on-track and ensuring postoperative shoulder stability [8]. These anatomical insights contribute to improved surgical outcomes and functional recovery [9].

Several studies have explored scapular morphology in diverse populations, including European, Turkish, Indian, and Chinese cohorts, reporting both similarities and marked differences [10–15]. These studies were conducted on patients, cadavers, or dried scapulae, using various measurement modalities such as magnetic resonance imaging (MRI), computed tomography (CT), and direct calliper-based assessments [16, 17]. However, data from North African and Middle Eastern populations remain limited. Population-specific anatomical data are essential in orthopaedic practice, as parameters such as glenoid width and coracoid dimensions directly influence prosthetic design, screw trajectory, and graft suitability [18].

Furthermore, methodological inconsistencies across studies have limited their clinical applicability. To address this gap, this study provides a comprehensive morphological and anthropometric analysis of dry scapulae from an Egyptian population using both three-dimensional CT-based and direct calliper-based measurements and compares these findings with those reported in other populations.

## Methods

This study was conducted in accordance with the Declaration of Helsinki and was approved by the Institutional Research Board of the authors’ affiliated institution. A total of sixty unpaired, dried, and preserved human scapulae of unknown age and sex were included. Paired specimens were not available. Only scapulae with intact anatomical landmarks and no signs of deformity were selected. A total of thirteen specimens were excluded due to fractures or deformities. Of the sixty included scapulae, thirty-two were right-sided and twenty-eight were left-sided. All specimens were sourced from the anatomy department of the institution, with academic use approved by the legal custodian of the bone collection. The identities of the specimens were anonymized, and respectful handling protocols were maintained throughout the study. Morphological evaluation was performed for the glenoid cavity, suprascapular notch, and acromial process. In addition, anthropometric measurements were obtained for all scapular components using both CT-based imaging and direct calliper-based methods.

### Morphological characteristics

**Fig. 1:**
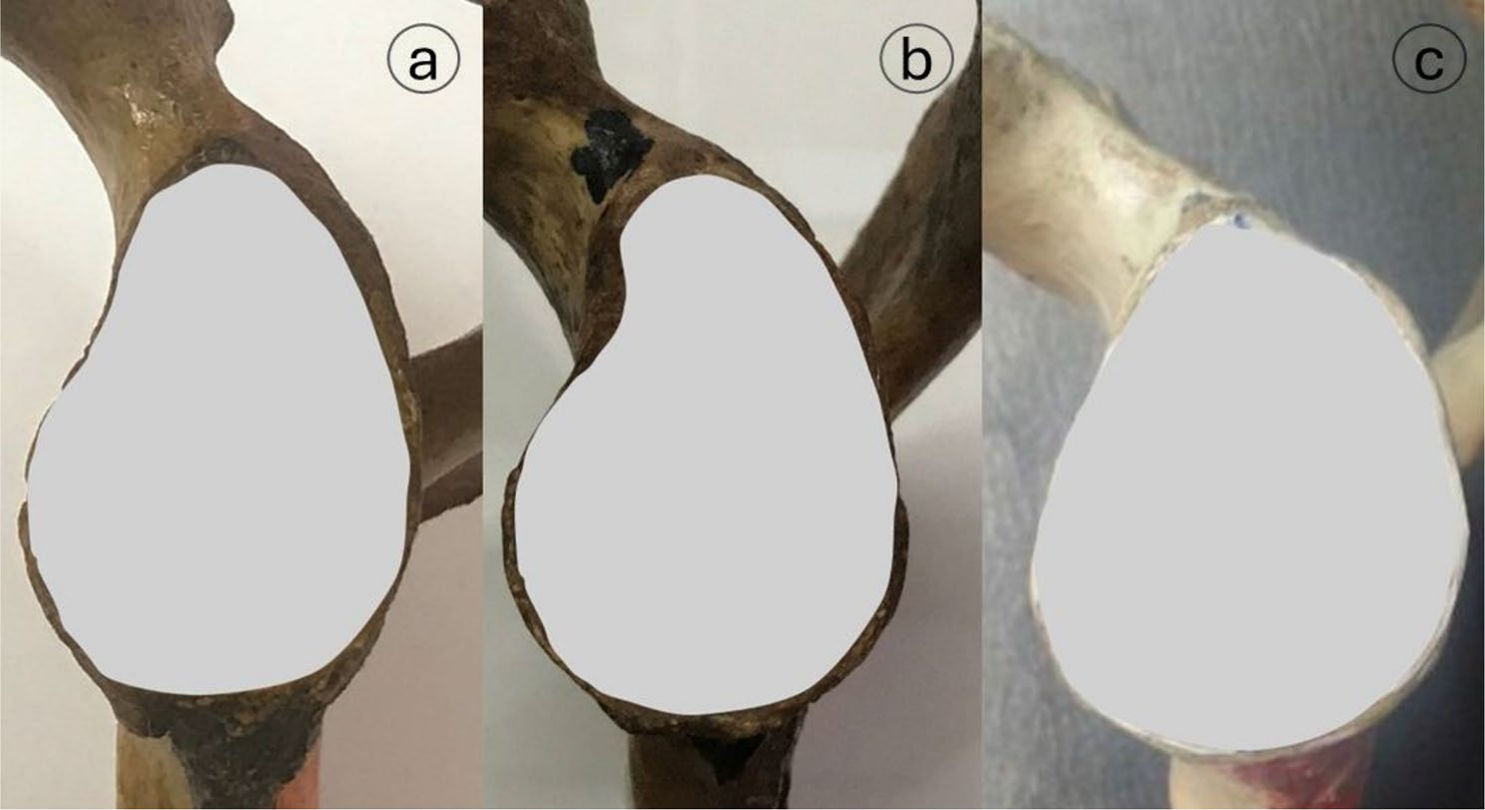
Representative glenoid cavity shapes: (**a**) pear-shaped, (**b**) inverted comma-shaped, and (**c**) oval-shaped

**Fig. 2:**
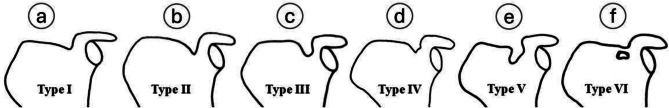
Classification of suprascapular notch morphology following Rengachary et al.: (**a**) type I, (**b**) type II, (**c**) type III, (**d**) type IV, (**e**) type V, (**f**) type VI (complete ossification forming a foramen)

**Fig. 3:**
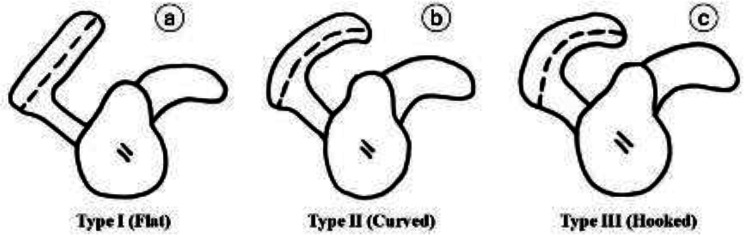
Bigliani classification of acromion morphology: (**a**) type I– flat, (**b**) type II – curved, (**c**) type III– hooked

The morphology of the glenoid cavity was described as oval, comma, or pear-shaped (Fig. 1). The morphology of the suprascapular notch was categorized using the **Rengachary et al.** classification [19], which divides the notch into Type I; is a wide depression that runs the entire length of the scapula, extending from the medial superior angle to the base of the coracoid process Type II is a wide, blunt, V-shaped notch; Type III is a symmetrical, u-shaped notch with parallel margins; Type IV is a small, V-shaped notch; Type V is similar to Type III in that the medial part of the ligament is ossified; and Type VI is a notch with the ligament completely ossified and forming a foramen (Fig. 2). Additionally, the acromial process morphology was classified by **Bigliani et al.** [20] into three types based on its slope (Fig. 3): type I (flat), type II (curved), and type III (hooked).

### Anthropometric evaluation

#### CT imaging interpretation

All specimens were scanned using a GE Optima CT520 system at the radiology department of our institution. The imaging protocol included a tube voltage of 120 kVp and a tube current of 300 mA. Axial images were acquired with a slice thickness of 0.9 mm and increment of 0.45 mm. The field of view (FOV) was set to 533 mm, with a matrix size of 512 × 512. These parameters ensured high-resolution imaging suitable for precise anatomical measurements. The images were reconstructed in three dimensions using the built-in workstation software and analyzed in the Digital Imaging and Communication in Medicine (DICOM) format. Measurements were performed using the multiplanar reconstruction (MPR) and volume-rendered (VR) views to ensure accuracy. Calibration was performed to ensure that linear measurements corresponded precisely to real dimensions.

Imaging interpretation and anatomical landmark identification were independently performed by two radiologists with over five years of experience in musculoskeletal imaging. Both radiologists were blinded to the results of the direct caliper measurements to avoid measurement bias. To evaluate measurement consistency, inter-rater reliability was assessed using intraclass correlation coefficients (ICCs) across all key parameters. All measurements were recorded and reported in millimeters (mm).

#### Direct calliper-based measurements

Direct anatomical measurements were obtained using a sliding Vernier caliper on each scapular specimen. Bones were handled with care, and each measurement was taken while ensuring full contact with the anatomical landmark and minimizing any angular deviation. All measurements were performed by a single investigator under standardized lighting and positioning conditions to ensure consistency. Each dimension was recorded twice, and the average value was used for analysis. Discrepancies exceeding 1 mm prompted remeasurement. Results were recorded in millimeters (mm).

### Glenoid cavity measurements

**Fig. 4:**
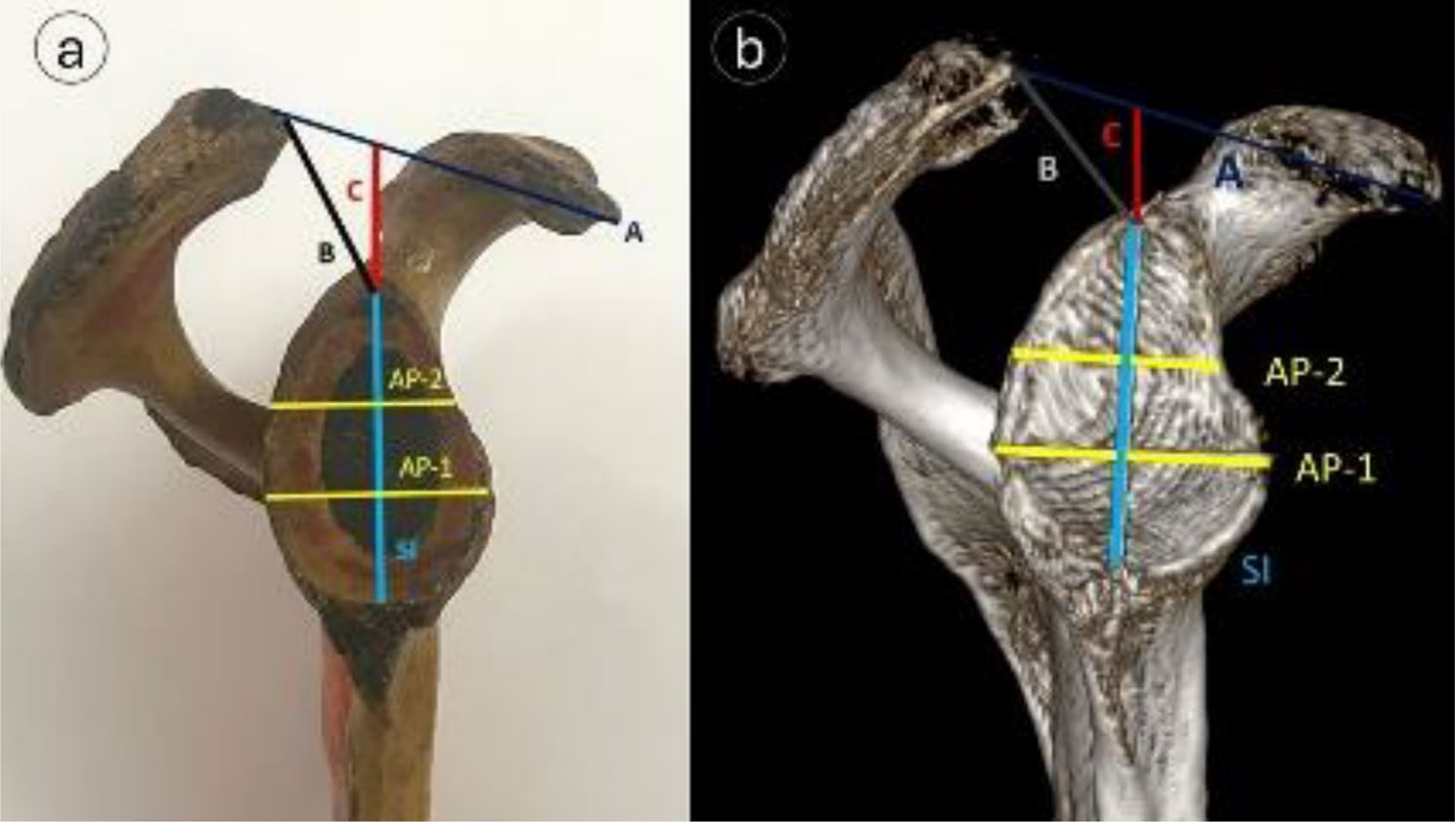
Morphometric analysis of glenoid and coracoacromial parameters: (**a**) dried scapular specimen, (**b**) corresponding 3D-CT image showing- A = Superior-Inferior glenoid diameter (light blue line), B = AP-1 diameter (yellow, lower half), C = AP-2 diameter (yellow, upper half), D = CA distance (dark blue), E = AG distance (black), F = CA arch interval (red)

Measurements were made of the Superior-Inferior (SI) and anterior-posterior diameters of the lower (AP-1) and upper (AP-2) glenoid cavities (Fig. 4). The greatest distance between the most conspicuous point of the supraglenoid tubercle and the inferior point on the glenoid margin was used to calculate the SI diameter. The maximum glenoid width perpendicular to the glenoid height was represented by the AP-1 diameter. The width of the upper glenoid half at the midpoint between the superior rim and the mid-equator was indicated by the AP-2 diameter.

### Scapular body measurements

**Fig. 5:**
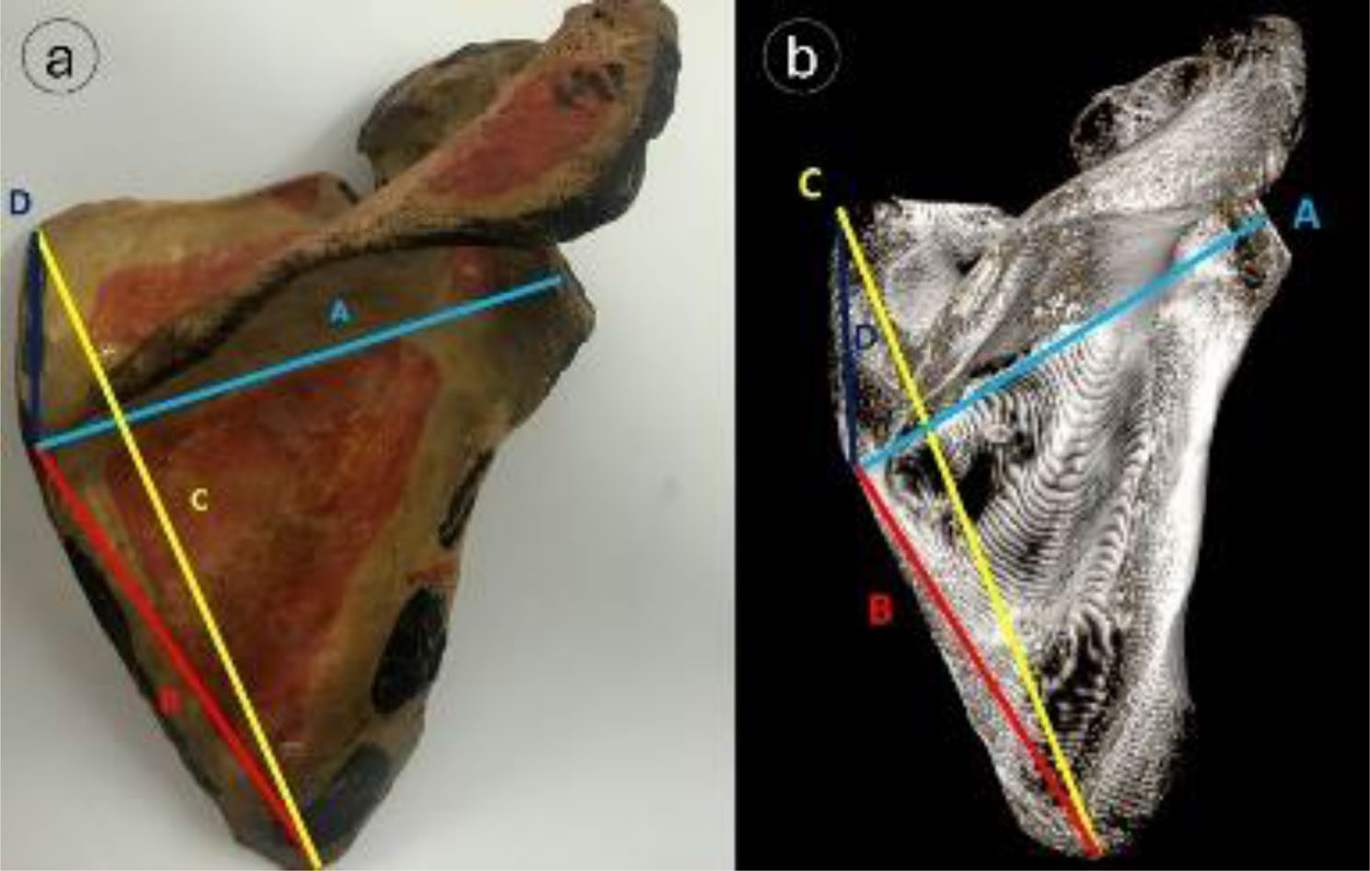
Scapular body dimensions (**a**) dried scapular specimen, (**b**) 3D-CT rendering with- A = Maximum scapular breadth (light blue line), B = infraspinatus length (red), C = maximum scapular length (yellow), D = supraspinatus length (dark blue)

The maximum scapular breadth, maximum scapular length, and infraspinatus length were measured. As illustrated in Fig. 5, the measurement of the scapular breadth was made from the point where the scapular spine intersects the medial border of the scapula to the middle of the outer glenoid cavity, The infraspinatus and supraspinatus lengths from the indicated point of intersection to the inferior and superior scapular angle’s tip, respectively. The maximum scapular length between the superior and inferior scapular angles. The ratio of the infraspinatus length to the maximum scapular length was used to calculate the glenoid index, which was then expressed as a percentage (%).

### Acromion process and acromion-related measurements

**Fig. 6:**
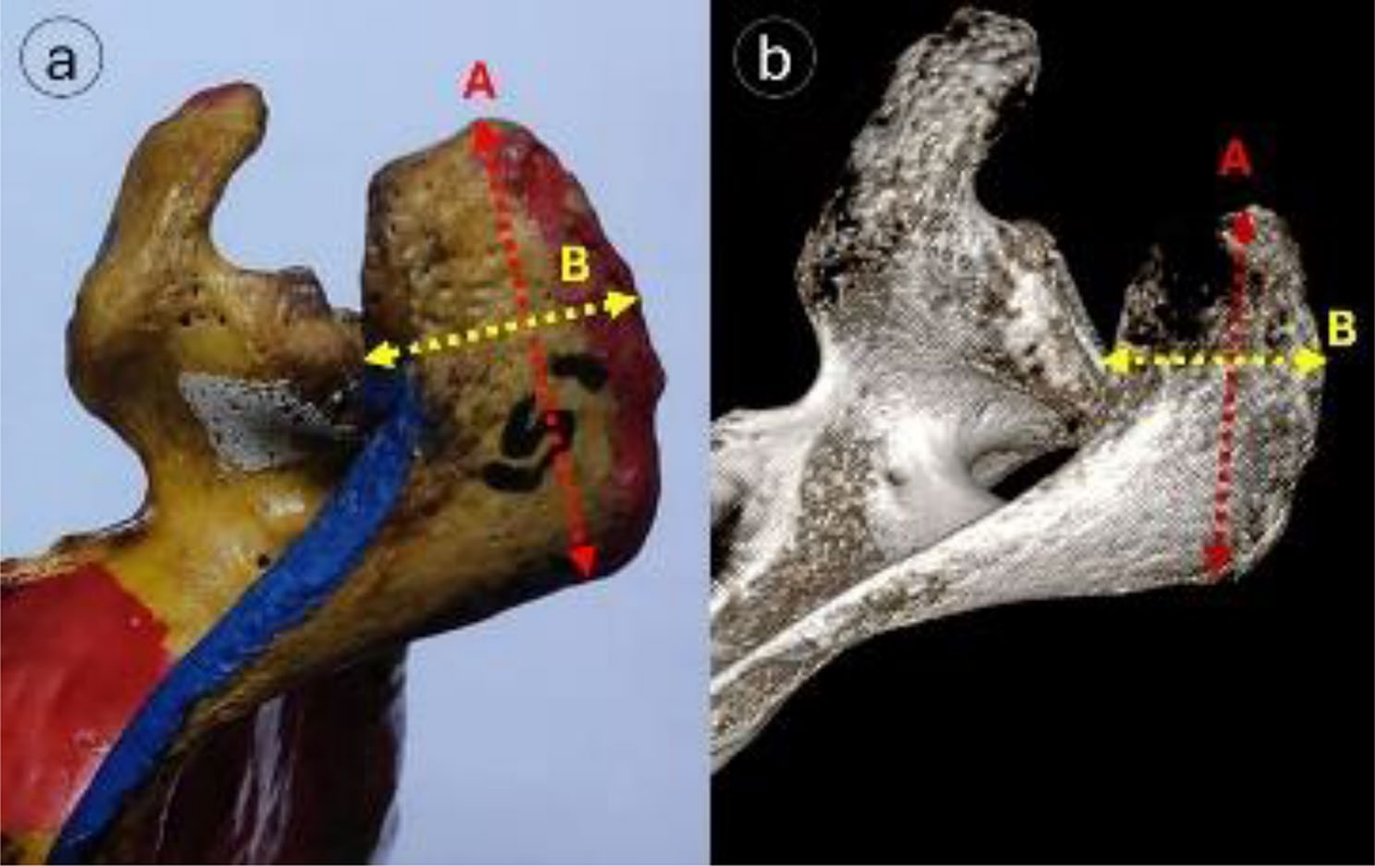
Acromion process measurements: (**a**) anatomical specimen, (**b**) 3D-CT reformatted image showing- A = Acromial length (red dotted line), B = acromial width (yellow dotted)

As illustrated in Fig. 6, the acromial width was measured at the mid-acromial point between the medial and lateral borders, the acromial thickness was thickness measured 10 mm behind the anterior border and 10 mm medial to the lateral border, and the acromial length was measured from its tip to the posterior border. Moreover, In Fig. 4, the coracoacromial (CA) distance demonstrated the distance between the tips of the acromion and coracoid processes. From the acromial tip to the supraglenoid tubercle, the Acromioglenoid (AG) distance was measured. The vertical distance from the supraglenoid tubercle to the CA plane was designated as the CA arch distance.

### Coracoid process and coracoid-related measurements

**Fig. 7:**
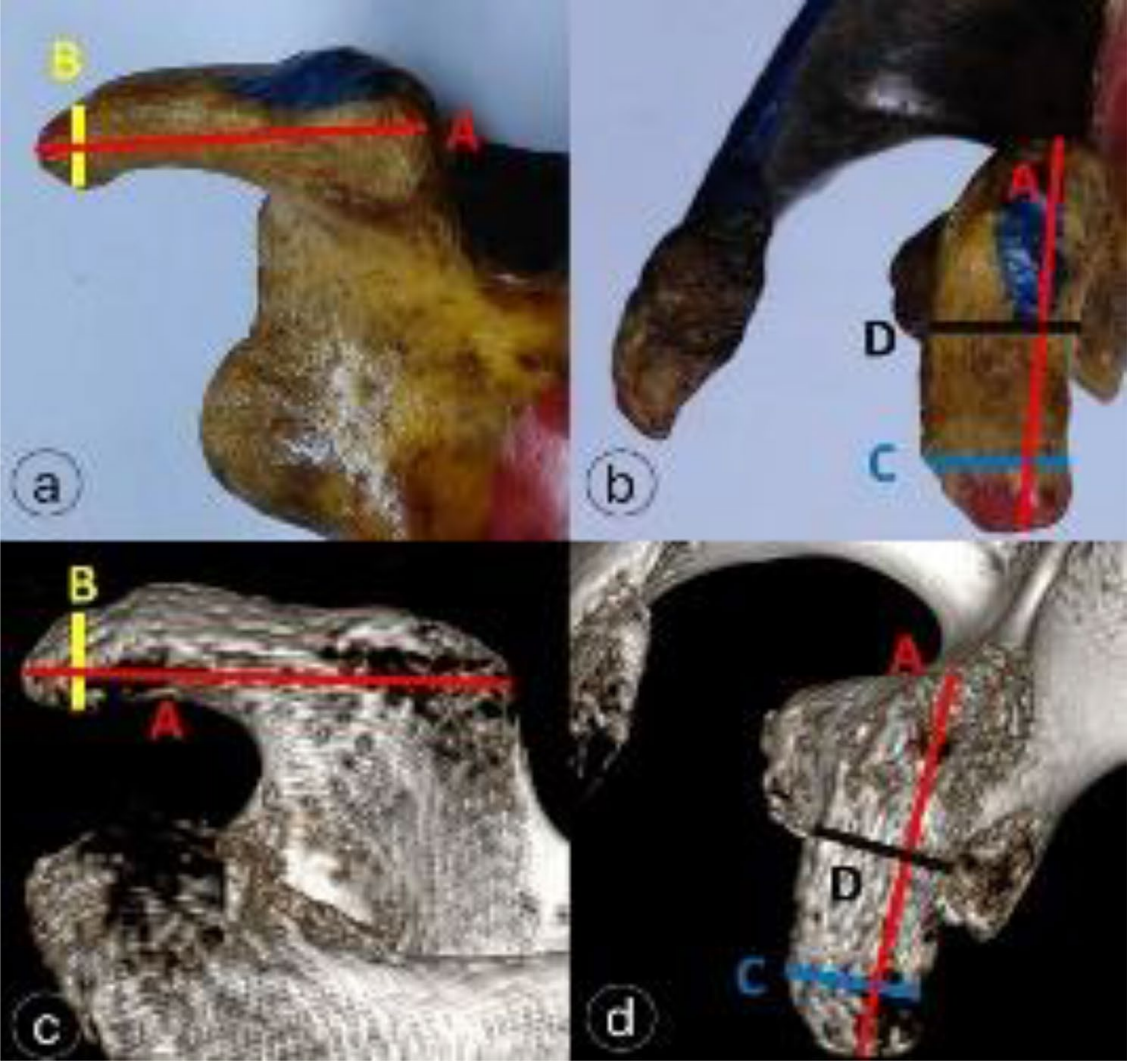
Coracoid morphometry across views: (**a**–**b**) dried specimens, (**c**–**d**) 3D-CT images indicating- A = coracoid length (red), B = tip thickness (yellow), C = tip width (light blue), D = base width (black)

**Fig. 8:**
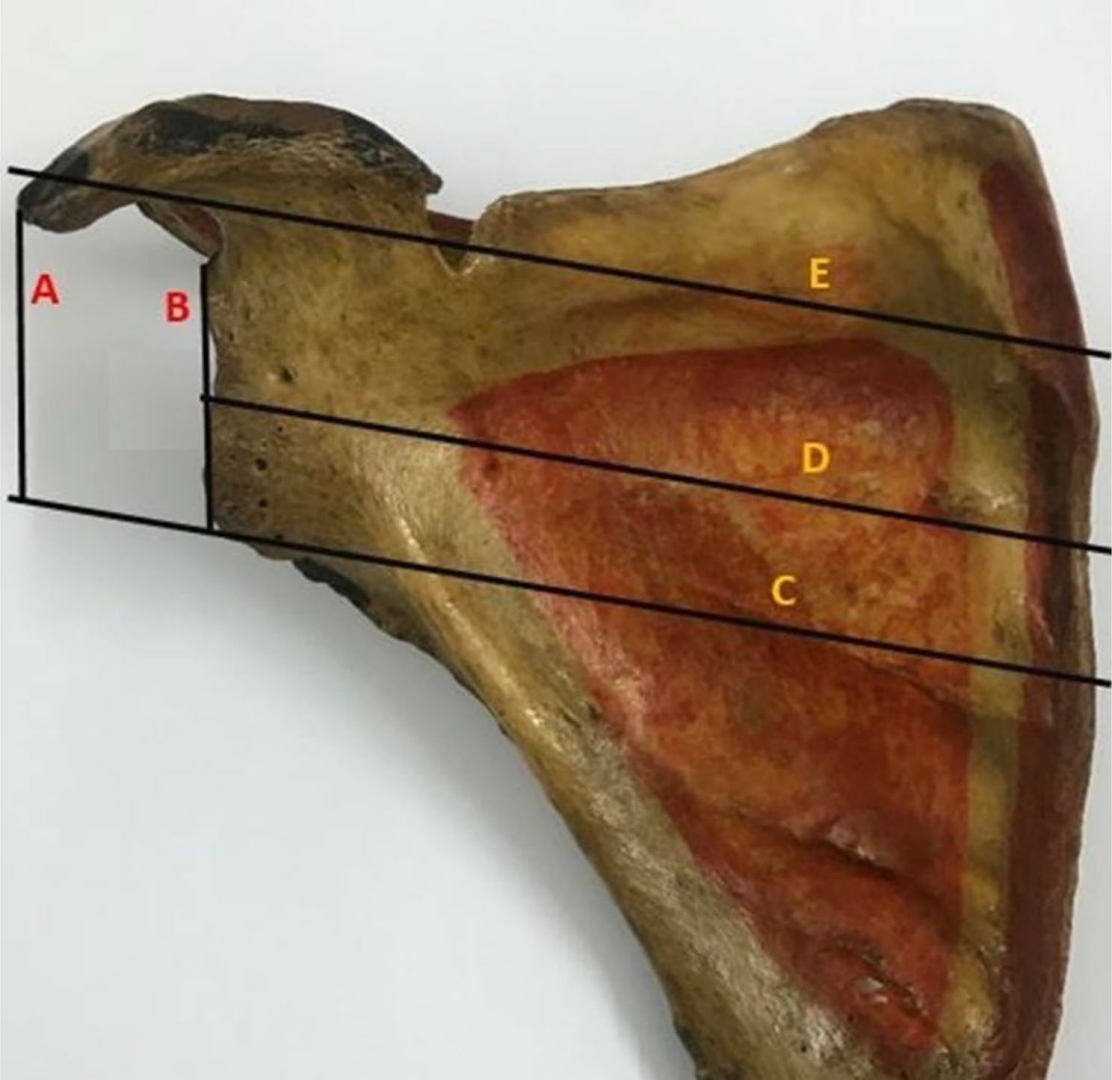
Measurement method for coracoid tip–inferior glenoid tubercle distance: (**A**) reference line between coracoid tip and tubercle, (**B**) vertical glenoid axis, (**C**–**D**) parallel planes for spatial orientation, (**E**) scapular spine axis [88]

Measurements were made of the coracoid process’s length (the distance between its tip and base), thickness, breadth, and distance, as well as the width of its base (Fig. 7). Furthermore, a line that cuts the coracoid tip and another that cuts the inferior glenoid tubercle—both of which are parallel to the scapular spine plane—were used to measure the distance between them in a plane parallel to the glenoid plane, termed the Coracoid tip-inferior glenoid tubercle distance (Fig. 8).

Comparative data with previously published studies have been included as Supplementary Tables 1–10 for reference and context.

### Statistical analysis

Data were analysed using IBM SPSS Corp. IBM SPSS Statistics for Windows, Version 22.0. Armonk, NY: IBM Corp. Numbers and percentages were used to describe the qualitative data. For non-parametric data, the median was used to characterize quantitative data. Standard deviation and mean are used to describe parametric data. Two independent groups with normally distributed and abnormally distributed data were compared using the unpaired t-test and the Man Witney U test. At the 0.05 level, the results’ significance was assessed.

## Results

On inspection, 50 out of the 60 scapulae exhibited a discernible glenoid notch. The glenoid cavity was oval in 50% of specimens (*n* = 30), pear-shaped in 45% (*n* = 27), and presented as an inverted comma shape in 5% (*n* = 3). According to the Rengachary et al. classification of the suprascapular notch, type III was the most common (35%, *n* = 21), followed by type IV (25%, *n* = 15), type I and type V (each 15%, *n* = 9), and type II (10%, *n* = 6). Regarding acromion morphology, type II was predominant (70%, *n* = 42), followed by type I (20%, *n* = 12) and type III (10%, *n* = 6).

CT-based measurements revealed a mean superior-inferior (SI) glenoid diameter of 35.7 ± 4.0 mm (range: 30–42 mm), an AP-1 diameter of 24.8 ± 2.0 mm (range: 20–30 mm), and an AP-2 diameter of 19.1 ± 2.0 mm (range: 15–24 mm). These values closely aligned with those obtained through direct calliper-based measurements. The glenoid index ranged from 50 to 85%, with a mean value of 70% ± 1%. The average coracoid tip–inferior glenoid tubercle distance was 35 ± 5 mm, and the mean acromial thickness was 7.6 ± 1.4 mm.

No statistically significant differences (*P* > 0.05) were observed between CT and direct measurements for any of the assessed parameters of the scapular body, acromion, or coracoid process (Table 1). However, a subgroup analysis comparing right- and left-sided unpaired scapulae identified significant differences in coracoid tip width and the coracoacromial (CA) arch interval, while all other variables showed no significant laterality-based variation (Table 2).

**Table 1:** Summary of scapular morphometric parameters obtained via CT-based and direct caliper-based methods

Measurement parameter	CT-based measurement	Direct measurement	*P*-value
	(Mean ± SD) (mm)		
**Glenoid cavity measurements**			
**SI diameter**	35.7 ± 4	36 ± 4	0.62
**AP-1 diameter**	24.8 ± 2	25.1 ± 2	0.61
**AP-2 diameter**	19.1 ± 2	19.3 ± 2	0.53
**Scapular body measurements**			
**Maximum length**	147.3 ± 4.2	147.8 ± 14.3	0.74
**Maximum width**	106.2 ± 14.3	107.9 ± 8.4	0.71
**Supraspinatus length**	44.3 ± 6.2	44.7 ± 6.1	0.65
**Infraspinatus length**	105.9 ± 19	109.1 ± 10	0.7
**Acromion process measurements**			
**Maximum length**	45 ± 6.6	45.1 ± 6.6	0.88
**Maximum width**	23.6 ± 3.9	23.8 ± 3.9	0.84
**Coracoid process measurements**			
**Maximum length**	43.4 ± 1.5	43.6 ± 1.8	0.54
**Base width**	15.6 ± 1.6	18.5 ± 2	0.33
**Tip thickness**	6.1 ± 0.9	6.3 ± 1	0.57
**Tip width**	13.9 ± 0.6	13.8 ± 0.7	0.59
**Acromion & Coracoid related measurements**			
**CA distance**	37.1 ± 6.3	37.3 ± 6.2	0.84
**AG distance**	28.1 ± 2.5	28.3 ± 2.5	0.74
**CA arch interval**	21 ± 4.3	21.8 ± 4.2	0.25

**Table 2:** Side-based comparison of morphometric features between right- and left-sided unpaired scapulae

Measurement parameter	CT-based measurement			Direct measurement		
	RT-sided (*n* = 32)	LT-sided (*n* = 28)	*P*-value	RT-sided (*n* = 32)	LT-sided (*n* = 28)	*P*-value
**Glenoid cavity measurements**						
**SI diameter**	35.2 ± 3.7	36.2 ± 4.3	0.42	35.5 ± 3.8	36 ± 4.3	0.41
**AP-1 diameter**	25 ± 2.7	24.5 ± 2.9	0.58	25.3 ± 2	24.8 ± 3	0.59
**AP-2 diameter**	19 ± 2	19 ± 2	0.65	19.3 ± 2	19.3 ± 1.9	0.88
**Scapular body measurements**						
**Maximum length**	145 ± 15	149 ± 12	0.18	145 ± 15	150 ± 12	0.18
**Maximum width**	106.4 ± 10	106 ± 17.9	0.15	106.5 ± 10	109 ± 4	0.07
**Supraspinatus length**	43.6 ± 6.9	45 ± 5.4	0.69	43 ± 6	45.6 ± 5	0.55
**Infraspinatus length**	106.3 ± 20	105 ± 19	0.93	109 ± 11	108 ± 8	0.86
**Acromion process measurements**						
**Maximum length**	44 ± 6.5	45 ± 6.8	0.95	45 ± 6.5	45 ± 6.9	0.99
**Maximum width**	23.8 ± 3.7	23.3 ± 4.1	0.65	23 ± 3.6	23 ± 4	0.68
**Coracoid process measurements**						
**Maximum length**	33 ± 1.6	33.7 ± 1.4	0.09	33.4 ± 1.5	33.7 ± 1.5	0.36
**Base width**	15.8 ± 1.4	15.3 ± 1.6	0.34	21 ± 2	15.7 ± 1.6	0.45
**Tip thickness**	6.1 ± 0.9	6.1 ± 0.9	0.89	6.2 ± 0.9	6.2 ± 0.9	0.84
**Tip width**	6.7 ± 0.4	7.2 ± 0.6	0.001^*^	6.7 ± 0.5	7.2 ± 0.6	0.008^*^
**Acromion & Coracoid related measurements**						
**CA distance**	38 ± 7.9	36.1 ± 3.5	0.77	38 ± 7	36.2 ± 3.6	0.71
**AG distance**	27.8 ± 2.7	28.6 ± 2.2	0.13	27 ± 2.7	28.8 ± 2.1	0.83
**CA arch interval**	18.8 ± 4	23.5 ± 2.8	< 0.0001^*^	19 ± 3.9	24.4 ± 2.9	< 0.0001^*^

Inter-rater agreement for CT-based measurements, assessed via ICCs, ranged from 0.87 to 0.96, demonstrating excellent reliability and consistency between the two independent radiologist readers. These findings confirm the robustness and reproducibility of the imaging-based morphometric approach.

## Discussion

Numerous studies across diverse populations have explored morphological variations of the scapula and its key bony landmarks, including the glenoid cavity, acromion, and coracoid process. These investigations have employed a range of samples, including patients, cadavers, and dried skeletal specimens [10, 13, 14], and have utilized various measurement techniques such as direct calliper assessments, CT, and MRI [12, 13, 15–17]. In the present study, a total of 60 unpaired, dried human scapulae were subjected to comprehensive anthropometric and morphological evaluation using both 3D-CT and direct measurement.

Rigorous specimen selection criteria were applied to ensure data integrity, with exclusion of any scapulae exhibiting visible deformities, preservation-related resorption, or pathological changes that could compromise anatomical accuracy. Each specimen underwent thorough inspection to confirm its suitability for inclusion in the study. By combining the precision of direct measurement with the advanced spatial resolution of 3D-CT imaging, this study sought to optimize both surface accuracy and internal landmark visualization. While calliper-based methods offer tactile precision for accessible structures, they are limited in reaching complex anatomical contours; conversely, 3D-CT provides broader spatial insight but may be influenced by resolution thresholds. This dual-modality approach was selected to mitigate individual method limitations and enhance the reliability of the dataset. Notably, no statistically significant differences were observed between the two measurement modalities across any of the parameters assessed, supporting the consistency and reproducibility of the methodology. The present findings demonstrate both congruencies and notable distinctions when compared to previously published reports.

The most consistent morphological feature in this study was the oval-shaped glenoid cavity, observed in 50% of the scapulae. This variant is characterized by the absence of a prominent glenoid notch along the anterior margin. Our findings are consistent with those of **Coskun et al.** [11] and **Nasr El-din & Ali** [21], who also reported the oval glenoid as the most common shape. In contrast, most other studies have identified the pear-shaped glenoid as the predominant configuration, followed by the inverted comma type [13, 14, 17, 22–25]. Notably, two additional reports found the pear-shaped cavity to be most prevalent, with the oval shape as the second most common [26, 27].

Understanding glenoid morphology is critical to shoulder biomechanics, as it directly affects joint stability and surgical planning. A distinct glenoid notch may compromise labral attachment along the anterior glenoid rim, increasing the risk of labral detachment during repetitive trauma. This predisposition can contribute to the development of Bankart lesions and subsequent anterior shoulder instability [26]. Recent investigations have also suggested a link between glenoid dimensions and joint stability, with taller, narrower glenoid (higher SI/AP ratios) being more susceptible to instability [28, 29].

Consistent with multiple studies [14, 19, 30–32], type III suprascapular notch was the most frequently observed notch morphology in our series (35%), followed by type IV (25%). This pattern is in line with the findings of **Senol et al.** [14]. and aligns partially with **Albino et al.** [10], who reported type IV as the most common. Other studies diverge in their rankings: **Sangam et al.** [32] and **Sinkeet et al.** [31] reported type I as the second most common, whereas **Natsis et al.** [30] and **Rengachary et al.** [19] identified type II in that position. Our finding that type VI was the least frequent notch type further supports patterns observed in prior literature.

The suprascapular notch is a critical anatomical landmark for the suprascapular nerve, particularly during shoulder arthroscopy [10]. The morphology of the notch plays a key role in guiding both diagnostic and interventional approaches. Bridged by the superior transverse scapular ligament, the notch can undergo partial or complete ossification, thereby narrow the passage and predispose the suprascapular nerve to compression or entrapment. Suprascapular nerve impingement syndrome accounts for approximately 1–2% of all cases of shoulder pain, and its occurrence has been most strongly linked to type III notches, due to their narrow, U-shaped configuration [33]. A thorough understanding of suprascapular notch variations is therefore crucial in preoperative planning, particularly for arthroscopic decompression and suprascapular nerve blocks [10].

In the present study, type II acromion was the most common morphology, identified in 70% of specimens. This finding aligns with several published studies across varied populations [11, 14, 15, 21, 30, 34–48]. Other authors, such as **Tangtrakulwanich & Kapkird** [49] and **Prasad et al.** [50], reported a predominance of type I acromion, whereas **Gupta et al.** [40] and **Singh et al.** [39] observed type III as the most frequent. Clinically, a hooked (type III) acromion is strongly associated with subacromial impingement, and RC tears. In such cases, acromioplasty is typically required to decompress the subacromial space and improve supraspinatus tendon mobility [51]. Both type II and III morphologies are considered risk factors for RC disease and may warrant additional acromioplasty, particularly in patients undergoing arthroscopic decompression or RC repair where fraying of the coracoacromial ligament is observed [52].

Considerable variation in the anthropometric scapular dimensions has been documented across prior studies. In the present study, the mean SI glenoid diameter was 36 ± 4 mm, which aligns closely with values reported in previous literature [11, 13, 25, 53–55]. However, several studies have documented lower mean SI diameters, particularly among females [22–24, 56, 57], while others have reported higher values [14, 16, 21, 58–64]. Among these, **DiStefano et al.** [60] recorded the highest mean SI glenoid diameter at 39.5 ± 2.6 mm, whereas **Hassanein** [24] reported the lowest, with 28.7 ± 4.1 mm in left-sided scapulae.

The mean AP-1 glenoid diameter in our study was 25 ± 2.8 mm, closely matching results from multiple earlier investigations [11, 14, 16, 24, 25, 54]. Some reports noted slightly lower values [13, 22, 23, 56, 62], while others found marginally higher measurements [21, 53, 55, 58–61, 63]. Similarly, the mean AP-2 diameter in this study was 19 ± 2.3 mm that was higher than values reported in several studies [13, 22, 23, 25] but lower than those found by **Senol et al.** [14]. and **Nasr Eldin & Ali** [21].

The assessment of glenoid dimensions is crucial for various surgical procedures, especially shoulder arthroplasty, where the placement of the glenoid base plate in either a total or reverse shoulder arthroplasty requires preoperative radiological planning of the bony glenoid to ensure that it is in line with the appropriate size of the glenoid base plate and determine whether additional augmentation of the glenoid is necessary [65]. Additionally, glenoid reaming and baseplate implantation can be guided by measuring the SI glenoid diameter. Before drilling and implanting the glenoid baseplate. **Matsen et al.** showed that the guidewire should be inserted into the glenoid at a point that is 13 mm anterior to the posterior glenoid rim and 19 mm superior to the inferior glenoid rim [18].

In the present study, the mean scapular length and width were 147 ± 14 mm and 107.9 ± 8.4 mm, respectively. These values are consistent with findings reported by **Aydemir et al.** [66] and **Nasr Eldin & Ali** [21]. Several studies reported greater length [14, 21, 67], while others observed shorter average values [13, 25, 56, 68–71]. Interestingly, the mean scapular width observed in our study exceeded that reported in most earlier investigations [13, 14, 25, 38, 56, 66–69, 71]. An exception was noted in a study by **Gosavi et al.** [70], which reported a markedly higher mean width of 141.4 mm.

The mean GI in our study was 70 ± 1%, aligning closely with values published by by **Tankala et al.** [13], **Senol et al.** [14], and **Parmar et al.** [72]. In contrast, **Ankushrao & Dombe** [25] reported a lower GI of 65.4 ± 8.1%. Conversely, higher averages were reported by **Hassanein** [24] at 73.6 ± 9% and **Polguj et al.** [73] at 72.5 ± 5.5%. These discrepancies may reflect population-based anatomical variation, measurement technique differences, or sample composition. Morphologically, the scapular body is recognized to exhibit modular forms; typically classified as straight, rounded, or wavy configurations [74]. Given that the RC musculature originates from the scapula, these structural variations, along with underlying anthropometric parameters, may influence shoulder biomechanics.

In line with two earlier reports [39, 75], the mean CA distance in the present study was 37.3 ± 6.2 mm. Several other studies reported lower average values for this parameter [15, 21, 38, 40, 43, 47, 48, 50, 76], whereas **Mansur et al.** [77] observed a comparatively higher mean CA distance. The mean AG distance measured in our study was 28.3 ± 2.5 mm, which is lower than the values reported by **Mansur et al.** [77], and **Akhtar et al.** [48], and **Vinay & Sivan** [43]. However, it is higher than the mean AG distances documented in a range of earlier studies [21, 38–40, 47, 50, 75]. Additionally, the mean CA arch interval in our sample was found to be 21.8 ± 4.2 mm.

Anatomically, shoulder impingement may arise due to compression at the level of the CA ligament, the anterior undersurface of the acromion, or both. Acromioplasty, commonly performed to treat subacromial impingement, involves the resection of the anterior acromial edge, the undersurface of the acromion, and the CA ligament to expand the subacromial space [78]. Preoperative evaluation of acromial thickness and the CA distance is therefore critical [47]. The CA ligament, which spans from the coracoid to the acromion, serves as a stabilizing structure that limits superior migration of the humeral head, particularly in the presence of massive RC tears. However, when this ligament becomes taut, particularly at its bony attachments, it can act as a constriction point, contributing to impingement symptoms. Arthroscopic release of the CA ligament in selected cases of impingement has been associated with significant postoperative pain relief [76].

The mean acromial length recorded in our study was 45 ± 6.6 mm, closely matching values reported by **Mansur et al.** [77], **Akhtar et al.** [48]., and **Singh et al.** [39]. In contrast, several studies have reported lower mean values [15, 40, 43, 45, 47, 50, 75, 76], while higher averages were observed by **Nasr El-din & Ali** [21], and **Paraskevas et al.** [38]. The average acromial breadth in our sample was 23.7 ± 3.8 mm. This value was lower than those reported by **Mansur et al.** [77], **Akhtar et al.** [48], **Vinay & Sivan** [43], and **Nasr El-din & Ali** [21], but exceeded the findings of **Paraskevas et al.** [38], **Panigrahi & Mishra** [75], and **Sinha et al.** [45]. Our results are consistent with those of **Thawanthorn & Chaimongkhol** [47], **Priya & Jain** [40, 76], and **Gupta et al.** [40], and **Singh et al.** [39].

The mean coracoid length in this study was 43.6 ± 1.8 mm, which is consistent with several earlier reports [12, 79–82]. Higher values were reported by **Knapik et al.** [83] and **Dolan et al.** [84], whereas lower values were observed in studies by **Imma et al.** [85] and **Kalra et al.** [57]. The average coracoid width was 18.5 ± 2 mm, exceeding those reported in previous literature [12, 57, 80, 84, 85]. Conversely, the mean coracoid tip thickness measured 6.3 ± 1 mm, which was slightly lower than values cited in most comparative studies [12, 57, 79–85]. The mean coracoid tip width in our study was 13.8 ± 0.7 mm, aligning closely with results from **Lian et al.** [80], **Jia et al.** [12], and **Fathi et al.** [79]. Meanwhile, lower values were noted by **Imma et al.** [85] and **Kalra et al.** [57], and higher measurements were reported by **Dolan et al.** [84], and **Terra et al.** [81], and **Knapik et al.** [83].

Preoperative planning for the Latarjet procedure in cases of recurrent shoulder instability benefits greatly from accurate coracoid morphometric assessment. Precise measurement of coracoid dimensions is essential for identifying the osteotomy site and ensuring that an adequate length of the coracoid can be transferred to the anteroinferior glenoid rim. This helps avoid intraoperative complications such as coracoid fracture during screw advancement, while also enabling proper screw length selection and positioning to promote optimal purchase, union, and implant stability [86, 87].

Supplementary Tables 1–10 summarize the key comparative morphometric findings from the current study alongside previously published data. Notably, the anatomical relationship between the coracoid tip and the inferior glenoid tubercle has received limited attention in the literature, despite its relevance for surgical orientation and imaging-based planning. In this study, the mean coracoid tip–inferior glenoid tubercle distance was 35 ± 5 mm. This relationship has implications for glenoid version assessment, which is typically measured on axial CT slices either at the level of the coracoid tip or the mid-axial glenoid plane, according to the Friedman method [65, 88]. Given the individual variability in the coracoid tip–glenoid alignment, the mid-axial glenoid level may offer a more consistent and practical reference point for version measurements in preoperative imaging [88].

The observed similarities and discrepancies between our scapular measurements and those reported in previous studies may be attributed to a combination of population-specific anatomical variation, methodological differences, and disparities in specimen preservation. Genetic, environmental, and lifestyle factors influence skeletal development and morphology, leading to distinct anatomical profiles across populations. Methodologically, prior research has employed a variety of measurement tools, ranging from direct to advanced imaging modalities, each with inherent limitations in accessibility and accuracy. By incorporating both direct and CT-based methods, our study aimed to mitigate these limitations and enhance the robustness of the dataset. Additionally, differences in preservation quality, including the degree of specimen degradation, may have impacted the precision of measurements in earlier studies. Collectively, these factors underscore the need for standardized, reproducible protocols in morphometric research to improve inter-study comparability and advance clinical application.

Population-specific anatomical variations in scapular morphology carry direct clinical implications, particularly in shoulder arthroplasty. For instance, glenoid width and height are critical for selecting an appropriately sized baseplate in total shoulder arthroplasty; under sizing or oversizing may compromise fixation and increase the risk of early loosening. In reverse shoulder arthroplasty, the morphology of the inferior glenoid becomes especially relevant. Ideally, the widest AP glenoid diameter should exceed the diameter of the smallest available baseplate to ensure stable fixation. Moreover, preoperative identification of glenoid wear often raises concern for bone grafting prior to baseplate implantation, to enhance implant stability, preserve joint biomechanics, and minimize the risk of early failure [65, 89, 90]. Recognizing these regional anatomical differences enhances patient-specific surgical planning and supports the design of prosthetic implants tailored to diverse populations.

These anatomical variations are equally relevant in non-arthroplasty shoulder procedures, such as the Latarjet procedure and fracture fixation. Accurate understanding of glenoid cavity dimensions is critical for evaluating bone loss in shoulder instability, particularly through 3D-CT imaging with glenoid en face reformats. Such imaging is pivotal in preoperative planning, helping to determine the suitability of arthroscopic Bankart repair versus bone block reconstruction. Coracoid size plays a key role in the feasibility of the Latarjet procedure; smaller coracoids may necessitate the use of alternative graft sources to ensure adequate screw fixation and avoid hardware complications [26, 65]. Therefore, the observed morphological differences in glenoid and coracoid anatomy have significant implications beyond academic interest. They are essential for advancing individualized treatment strategies and optimizing outcomes in shoulder surgery.

While previous studies have assessed scapular morphology, most have focused on Asian or European populations, often using a single measurement modality. This study addresses a significant gap by offering a dual-modality analysis in a North African population, integrating both CT imaging and direct anatomical measurements. These findings add novel, population-specific data to the existing literature and may be especially useful in adapting surgical and radiological practices to underserved or understudied populations. In this way, the study not only reinforces earlier work but also extends it in clinically meaningful ways.

This study has several limitations that must be acknowledged. Firstly, the scapulae analyzed were unpaired, which precluded side-to-side comparison and limited the investigation of bilateral anatomical asymmetry. Such comparisons could have provided further insights into natural morphological variability. Secondly, the exclusion of deformed or damaged scapulae, although necessary to ensure measurement accuracy, may have introduced selection bias. This limits the generalizability to broader clinical populations. Thirdly, demographic data such as age, sex, and body size of the specimens were not available. This precludes subgroup analysis based on these important variables, which are known to influence scapular morphology. Lastly, the relatively small sample size, although comparable to previous studies, may reduce the statistical power to detect subtle anatomical differences. Future studies with larger, paired samples and known demographic profiles are recommended to validate and expand upon these findings.

## Conclusion

The scapular bony components demonstrate notable interpopulation variability in both morphological features and anthropometric measurements. In this study, 3D-CT imaging and direct calliper-based assessments yielded closely aligned results, underscoring the reliability of CT as a practical tool for preoperative evaluation. Accurate measurement of scapular anatomy is essential for optimizing surgical planning and execution. A thorough understanding of glenoid, acromial, and coracoid morphology enhances clinical decision-making in procedures such as glenoid baseplate implantation during shoulder arthroplasty and screw placement in the Latarjet procedure. These findings reinforce the value of population-specific anatomical data in improving surgical precision and outcomes in shoulder interventions.

## Electronic supplementary material

Below is the link to the electronic supplementary material.

Supplementary Material 1

## Data Availability

All data generated or analysed during this study are included in this published article and its supplementary information file.
