# Supplementary material for: Comprehensive analysis of bony scapula morphology and anthropometry in a homogeneous population
Source: BMC Med Imaging. 2025 Jul 2;25:262. doi: 10.1186/s12880-025-01793-z (PMC12225530; doi:10.1186/s12880-025-01793-z)
Supplement: Supplementary file 1 — Supplementary Material 1 [file 12880_2025_1793_MOESM1_ESM.docx]

**Supplementary Tables 1–10**

**Comparative morphometric data from previously published studies**

***Corresponding to the manuscript: “Comprehensive Analysis of Bony Scapula Morphology and Anthropometry in a Homogeneous Population***

**Supplementary Table 1.** Frequency distribution of glenoid cavity shapes versus previous studies.

| **Study, year** | **Glenoid cavity morphology (%)** | | |
| --- | --- | --- | --- |
|  | **Pear** | **Inverted comma** | **Oval** |
| **Present study** | 45% | 5% | 50% |
| **Tankala et al. [13], 2023** | 48.61% | 34.62% | 17.36% |
| **Senol et al. [14], 2023** | 87.5% | 4.16% | 8.3% |
| **Ülkir et al. [27], 2022** | 71.4 % | 2.5 % | 26.1 % |
| **Raaj et al. [17], 2018** | Right 56%  Left 52% | Right 36%  Left 32% | Right 8%  Left 16% |
| **Ankushrao & Dombe [25], 2017** | 53.27% | 32.71% | 14.01% |
| **Hassanein [24], 2015** | 45.59% | 30.88% | 23.53% |
| **Nasr El-din & Ali [21], 2015** | 31.25% | 18.12% | 50.63% |
| **Rajput et al. [23], 2012** | Right 49%  Left 46% | Right 35%  Left 39% | Right 16%  Left 15% |
| **Mamatha et al. [22], 2011** | Right 46%  Left 43% | Right 34%  Left 33% | Right 20%  Left 24% |
| **Coskun et al. [11], 2006** | 28% | 0% | 72% |
| **Prescher & Klumpen [26], 1997** | 55% | 0% | 45% |

**Supplementary Table 2.** Comparative analysis of suprascapular notch types across studies

| **Study, year** | **Suprascapular notch morphology (%)** | | | | | |
| --- | --- | --- | --- | --- | --- | --- |
|  | **Type I** | **Type II** | **Type III** | **Type IV** | **Type V** | **Type VI** |
| **Present study** | 15% | 10% | 35% | 25% | 15% | 0% |
| **Senol et al. [14], 2023** | 12.5% | 16.6% | 37.5% | 33.3% | 0% | 0% |
| **Albino et al. [10], 2013** | 12.4% | 19.8% | 22.8% | 31.1% | 10.2% | 3.6% |
| **Sangam et al. [32], 2013** | 21.5% | 8.65% | 59.61% | 2.88% | 5.76% | 1.93% |
| **Sinkeet et al. [31], 2010** | 22% | 21% | 29% | 5% | 18% | 4% |
| **Natsis et al. [30], 2007** | 8% | 31% | 48% | 3% | 6% | 4% |
| **Rengachary et al. [19], 1979** | 6% | 24% | 40% | 13% | 11% | 6% |

**Supplementary Table 3.** Incidence of acromion process types: current findings versus earlier reports.

| **Study, year** | **Acromion morphology** | | |
| --- | --- | --- | --- |
|  | **Type I (%)** | **Type II (%)** | **Type III (%)** |
| **Present study** | 20% | 70% | 10% |
| **Akhtar et al. [48], 2023** | 28.33% | 53.34% | 18.33% |
| **Senol et al. [14], 2023** | 33.3% | 57.14% | 9.5% |
| **Koca et al. [15], 2022** | 21% | 62% | 17% |
| **Thawanthorn & Chaimongkhol [47], 2020** | 9.2% | 78.8% | 12% |
| **Alraddadi et al. [46], 2019** | 2% | 55% | 43% |
| **Prasad et al. [50], 2019** | 57.14% | 40% | 2.85% |
| **Guo et al. [44], 2018** | 47.3% | 50% | 3% |
| **Sinha et al. [45], 2018** | 24.59% | 49.18% | 26.22% |
| **Vinay & Sivan [43], 2017** | 37.1% | 47.5% | 15.2% |
| **Schetino et al. [42], 2017** | 5.6% | 57.89% | 36.84% |
| **Nasr El-din & Ali [21], 2015** | 26.9% | 45.62% | 15% |
| **Naidoo et al. [41], 2015** | 34.6% | 51.1% | 14% |
| **Gupta et al. [40], 2014** | 32% | 22% | 46% |
| **Singh et al. [39], 2013** | 22.5% | 38.8% | 38.8% |
| **Tangtrakulwanich & Kapkird [49], 2012** | 84.5% | 10.7% | 4.8% |
| **Paraskevas et al. [38], 2008** | 26.1% | 55.6% | 18.1% |
| **Nastsis et al. [30], 2007** | 12.1% | 56.5% | 28.8% |
| **Sangiampong et al. [37], 2007** | 3.2% | 93.5% | 3.2% |
| **Coskun et al. [11], 2006** | 10% | 73% | 17% |
| **Getz et al. [35], 1996** | 22.8% | 68.5% | 8.6% |
| **Nicholson et al. [36], 1996** | 32% | 42% | 26% |
| **Banas et al. [34], 1995** | 39% | 51% | 10% |

**Supplementary Table 4.** Cross-study comparison of SI- and AP-1 glenoid diameters.

| **Study, year** | **Glenoid cavity diameters (Mean ± SD) (mm)** | |
| --- | --- | --- |
|  | **SI diameter** | **AP-1 diameter** |
| **Present study** | CT-based: 35.7±4  Direct: 36±4 | CT-based: 24.8±3  Direct: 25±3 |
| **Senol et al. [14], 2023** | Total 37.9±0.31  Right 37.7±3  Left 38.5±3.4 | Total 24.7±0.27  Right 24.1±2.2  Left 25.8±3.2 |
| **Tankala et al. [13], 2023** | Right 36.03±2.15  Left 35.52±2.12 | Right 22.67±1.53  Left 22.59±1.47 |
| **Ülkir et al. [64], 2022** | Male 38.54±2.61  Female 34.04±2.21 | Male 27.83±2.69  Female 23.77±2.14 |
| **Mathews et al. [53], 2017** | 36.6 | 27.8 |
| **Ankushrao & Dombe [25], 2017** | 36.71±4.14 | 24.85±3.50 |
| **Kalra et al. [57], 2016** | 34 ± 3.6 | 23.9 ± 3.2 |
| **Nasr El-din & Ali [21], 2015** | Total 38.88±2.63  Right 38.88±2.63  Left 39.01±2.49 | Total 28.15±2.58  Right 28.31±2.38  Left 27.99±2.55 |
| **Hassanein [24], 2015** | Right 33.1 ± 3.9  Left 28.7 ± 4.1 | Right 24.4±2.21  Left 22.1±4.4 |
| **Patel et al. [56], 2013** | Male 34.6±3.5  Female 31.2±1.6 | Male 23.8±2.2  Female 22.2 ±2.7 |
| **Rajput et al. [23], 2012** | Right 34.76±3  Left 34.43±3.21 | Right 23.31±3.0  Left 22.92±2.80 |
| **DiStefano et al. [60], 2011** | 39.5 ± 2.6 | 31 ± 2.5 |
| **Mamatha et al. [22], 2011** | Right 33.67±2.82  Left 33.92±2.87 | Right 23.35±2.04  Left 23.02±2.30 |
| **Karelse et al. [55], 2007** | 35.9±3.6 | 27.2±3 |
| **Ozer et al. [59], 2006** | Male 38.71±2.71  Female 33.79±3.08 | Male 27.33±2.4  Female 22.72±1.72 |
| **Coskun et al. [11], 2006** | 36.3±3 | 24.6±2.5 |
| **Kwon et al. [16], 2005** | 37.8 | 26.8 |
| **Piyawinijwong et al. [58], 2004** | Male 38.1±2.2  Female 33.6±3.0 | Male 29.1±2.6  Female 25.6±2.5 |
| **Frutos [54], 2002** | Male 36.08± 2.0  Female 31.17± 1.7 | Male 26.31± 1.5  Female 22.31± 1.4 |
| **Checroun et al. [61], 2002** | 37.9 | 29.3 |
| **Churchill et al. [62], 2001** | 37.5 | 23.6 |
| **Iannotti et al [63], 1992** | 39 | 29 |

**Supplementary Table 5.** Comparative values of AP-2 glenoid diameters across populations.

| **Study, year** | **AP-2 glenoid diameter**  **(Mean ± SD) (mm)** |
| --- | --- |
| **Present study** | CT- based: 19±2  Direct: 19±2 |
| **Senol et al. [14], 2023** | 20.1±0.31 |
| **Tankala et al. [13], 2023** | Right 17.3±1.16  Left 17±1.34 |
| **Ankushrao & Dombe [25], 2017** | 16.31±3.16  16.2±3.64 |
| **Nasr El-din & Ali [21], 2015** | 21.51±2.69 |
| **Rajput et al. [23], 2012** | Right 16.2±3.23  Left 15.24±2 |
| **Mamatha et al. [22], 2011** | Right 16.21±2.1  Left 15.77±1.96 |

**Supplementary Table 6.** Comparative assessment of scapular length and width across earlier investigations

| **Study, year** | **Maximum scapular length** | **Maximum scapular width** |
| --- | --- | --- |
|  | **(Mean ± SD) (mm)** | |
| **Present study** | CT-based: 147.3 ± 4.2  Direct: 147.8±14.3 | CT-based: 106.2 ± 14.3  Direct: 107.9±8.4 |
| **Ülkir [64], 2023** | Male 159.13±12.20  Female 141.71±9.43 | Male 106.79±6.26  Female 96.38±6.28 |
| **Senol et al. [14], 2023** | 150.2 | 104 |
| **Tankala et al. [13], 2023** | 135.7±12.85  134.11±12.9 | 98.12±7.87  97.28±8.12 |
| **Aydemir [66], 2020** | 147 | 105 |
| **Ankushrao & Dombe [25], 2017** | 136.07±14.13 | 97.13±10.63 |
| **Lingamdenne et al. [68], 2016** | 141.49±9.74 | 98.69±6.98 |
| **Costa et al. [67], 2016** | Male 151.143  Female 132.63 | Male 102.43  Female 90.81 |
| **Nasr El-din & Ali [21], 2015** | 151.16±10.32 | 107.22±9.74 |
| **Chhabra et al. [69], 2015** | 98.69 | 103.65 |
| **Gosavi et al. [70], 2014** | 123.02 | 141.4 |
| **Patel et al. [56], 2013** | Male 136±11.4  Female 119.6±8.8 | Male 100.6±8.5  Female 93.5±4.3 |
| **Singhal et al. [71], 2013** | 141.7±8.9 | 96.4±7 |
| **Paraskevas et al. [38], 2008** | 147.6 | 101.9 |

**Supplementary Table 7.** Glenoid index values: current study versus previously published data.

| **Study, year** | **Glenoid index (Mean ± SD) (%)** |
| --- | --- |
| **Present study** | 70±1 |
| **Tankala et al. [13], 2023** | Right 68.44±7.98  Left 68.84±7.64 |
| **Senol et al. [14], 2023** | 69.74±5.8 |
| **Parmar et al. [72], 2017** | 69.09 |
| **Ankushrao & Dombe [25], 2017** | 65.40±8.14 |
| **Hassanein [24], 2015** | Right 73.67±9.08  Left 76.71±8.73 |
| **Polguj et al. [73], 2011** | 72.35±5.55 |

**Supplementary Table 8.** Cross-population comparison of CA- and AG- distances.

| **Study, year** | **CA distance** | **AG distance** |
| --- | --- | --- |
|  | **(Mean ± SD) (mm)** | |
| **Present study** | CT-based: 37.1±6.3  Direct: 37.3±6.2 | CT-based: 28.1±2.5  Direct: 28.3±2.5 |
| **Akhtar et al. [48], 2023** | Right 34.59 | Right 32.31 |
|  | Left 37.46 | Left 33.18 |
| **Koca et al. [15], 2022** | 30.48 | - |
| **Thawanthorn & Chaimongkhol [47], 2020** | 29.72 | 25.96 |
| **Priya & Jain [76], 2020** | 33.35 | 29.41 |
| **Prasad et al. [50], 2019** | 30.90 | 24.90 |
| **Panigrahi & Mishra [75], 2018** | Right 37.49 | Right 26.39 |
|  | Left 37.23 | Left 24.20 |
| **Sinha et al. [45], 2018** | 35.94 | 28.28 |
| **Vinay & Sivan [43], 2017** | Right 33.81 | Right 29.79 |
|  | Left 34.34 | Left 30.36 |
| **Nasr El-din & Ali [21], 2015** | 31.34 | 27.39 |
| **Gupta et al. [40], 2014** | Right 31.80 | Right 25.30 |
|  | Left 30.30 | Left 24.30 |
| **Mansur et al. [77], 2013** | Right 26.63 | Right 31.00 |
|  | Left 39.39 | Left 31.97 |
| **Singh et al. [39], 2013** | 37.50 | 27.00 |
| **Paraskevas et al. [38], 2008** | 28.10 | 17.70 |

**Supplementary Table 9.** Morphometric evaluation of the acromion process across previous studies.

| **Study, year** | **Side** | **Acromial length** | **Acromial breadth** | **Acromial thickness** |
| --- | --- | --- | --- | --- |
|  |  | **(Mean ± SD) (mm)** | | |
| **Present study** | | CT: 45 ± 6.6  Direct: 45.1±6.6 | CT: 23.6 ± 3.9  Direct: 23.8±3.9 | 7.6±1.4 |
| **Akhtar et al. [48], 2023** | Right | 44.52 | 28.31 | 7.10 |
|  | Left | 45.13 | 28.34 | 7.53 |
| **Koca et al. [15], 2022** | | 36.21 | - | - |
| **Thawanthorn & Chaimongkhol [47], 2020** | | 43.55 | 24.52 | 8.53 |
| **Priya & Jain [76], 2020** | | 41.65 | 24.50 | 7.62 |
| **Prasad et al. [50], 2019** | | - | - | - |
| **Panigrahi & Mishra [75], 2018** | Right | 41.72 | 21.42 | 6.68 |
|  | Left | 38.97 | 21.57 | 6.59 |
| **Sinha et al. [45], 2018** | | 41.23 | 22.12 | 7.01 |
| **Vinay & Sivan [43], 2017** | Right | 42.48 | 26.68 | - |
|  | Left | 42.46 | 26.44 | - |
| **Nasr El-din & Ali [21], 2015** | | 52.81 | 32.05 | 9.06 |
| **Gupta et al. [40], 2014** | Right | 41.60 | 23.20 | 7.30 |
|  | Left | 42.50 | 24.90 | 7.40 |
| **Mansur et al. [77], 2013** | Right | 46.46 | 26.63 | - |
|  | Left | 45.57 | 27.23 | - |
| **Singh et al. [39], 2013** | | 46.10 | 23.20 | 6.60 |
| **Paraskevas et al. [38], 2008** | | 46.10 | 22.30 | 8.80 |

**Supplementary Table 10.** Comparative morphometry of the coracoid process: present study vs. literature

| **Study, year** | **Coracoid length** | **Coracoid width** | **Tip thickness** | **Tip width** |
| --- | --- | --- | --- | --- |
|  | **(Mean ± SD) (mm)** | | | |
| **Present study** | CT: 43.4 ± 1.5  Direct: 43.6±1.8 | CT: 15.6 ± 1.6  Direct: 18.5±2 | CT: 6.1 ± 0.9  Direct: 6.3±1 | CT: 13.9 ± 0.6  Direct: 13.8 ± 0.7 |
| **Jia et al. [12], 2020** | 41.6 ± 4 | 14.5 ± 2 | 9 ± 1.8 | 13 ± 2 |
| **Knapik et al. [83], 2018** | 46 ± 3.7 | – | 9.2 ± 1.2 | 15.9 ± 1.9 |
| **Fathi et al. [79], 2017** | 42.4 ± 1 | – | 9 ± 0.5 | 13.1± 0.5 |
| **Imma et al. [85], 2017** | 37.9 ± 4.3 | 13.8 ± 1.7 | 9.2 ± 1.1 | 11.6 ± 2 |
| **Kalra et al. [57], 2016** | 40.4±4.4 | 14.1±2.3 | 8.5±1.7 | 10.3±2.3 |
| **Lian et al. [80], 2016** | 42.1 ± 2.3 | 15.2 ± 1.7 | 9.1 ± 1.7 | 13.6 ± 2 |
| **Terra et al. [81], 2013** | 42.6 ± 2.6 | – | 14.9 ± 1.2 | 21.1 ± 2 |
| **Dolan et al. [84], 2011** | 45.6 ± 4.2 | 16.1 ± 2.3 | 11.5 ± 0.9 | 18.3 ± 1.8 |
| **Salzmann et al. [82], 2008** | 43.1 ± 2.2 | – | 8.2 ± 1 | 13.6 ± 2.1 |
